# Supplementary material for: Standardising Training of Nurses in an Evidence-Based Psychosocial Intervention for Perinatal Depression: Randomized Trial of Electronic vs. Face-to-Face Training in China
Source: Int J Environ Res Public Health. 2022 Mar 30;19(7):4094. doi: 10.3390/ijerph19074094 (PMC8998312; doi:10.3390/ijerph19074094)
Supplement: Supplementary file 1 [file ijerph-19-04094-s001.zip › ijerph-1619950-supplementary.pdf]

Table S1: Enhancing Assessment of Common Therapeutic factors (ENACT) (adapted for WHO Thinking Healthy Program)

**Item1: Developing a bond without verbal communication and listening empathically: Eye contact, facial expressions and body language**<br>**1=Needs improvement**=No eye contact with the mother or staring constantly, expressing anger, mocking at her, interrupting the mother repeatedly, ignoring her, talking on phone without asking for permission.<br>**2=Done partially**=showing consistent lack of interest through body language, rare eye contact, not expressing emotions, pretence<br>**3= Done well** = Proper eye contact with the mother during conversation/discussion, smiling according to situation, proper seating arrangement and showing interest by leaning towards mother, and listening attentively using hmm…yes, or any other local expression.
**Item2: Communication skills: Asking open ended questions, summarizing and explaining the discussion.**<br>**1=Needs improvement**=asking close ended questions for example, will you do this? Can you do this?<br>**2=Done partially**=asking open ended questions but not going in detail and not asking for mother's opinion after summarizing the discussion.<br>**3=Done well**=asking open-ended questions, summarizing the discussion and asking the details, for example, what happened? Tell me more.
**Item 3:Developing a relationship (when the other person feels comfortable in talking to you) and introducing yourself.**<br>**1=Needs improvement**=Student does not introduce herself or make the mother feel comfortable or Student talks mostly about her personal experiences during the session.<br>**2=Done partially**=Student introduces herself but does not make the conversation comfortableforthemotherbychitchatorthestudenttalksaboutherselftothemotherbuti thasno link with mother's situation.<br>**3=Done well**=Student introduces herself, tries to make the conversation easy for the mother and shares her personal experiences relating to mother's situation/condition.
**Item4:Indepth understanding of the matter, explaining and telling her that such emotions can be felt often.**<br>**1=Needs improvement**=Student does not talk about mothers feelings or makes her own judgment or criticizes mother's feelings (for example, you should not think that, you should stop thinking and feeling about it)

**2=Done partially**=Student asks about mother's feelings and emotions but does not empathize with her, agree with her feelings or ask for details.<br>**3=Done well**=Student explains mother's feelings by relating them to the current situations and if appropriate, tells her that such emotions can be felt during these situations.
**Item5: Dealing with empathy, warmth and sincerity (with being pretentious)**<br>**1=Needs improvement** = Student criticizes mother's concerns and complaints or behaves angrily or rejects her views.<br>**2=Done partially**=Generally, Student's attitude is warm and friendly but she does not have the ability to empathize with her/relate with mother's perspective<br>**3=Done well**=Student expresses that she understands hat mother feels exactly how mostly people feel during this situation.
**Item6: Viewing daily activities and effects on life**<br>**1=Needs improvement**=Student does not ask mother about her thoughts, feelings and psychological issues and their effects on her life<br>**2=Done partially**=Student asks about daily activities and tasks but does not link it to mental or psychological problems<br>**3=Done well**=Student talks about the connection between psychological problems and daily activities
**Item7:Knowing about what explanations (simple/common and explanatory model) do the mother and her social support (family members and friends) give about mother's problems.**<br>**1=Needs improvement**=Student does not ask the mother about reasons of her problems, or she makes her own judgment, criticizes mother when she gives any explanation (for example, evil eye or black magic are not the cause of your problems, this is an ignorant and orthodox way of thinking.<br>**2=Done partially**=Student asks them other about reasons of her problems but does not probe further if her family members also think the same way? (for example, when in introductorysessiontwosidesofapicturesareshowntoknowtheperceptionoffamilymembers)<br>**3=Done well=**Student asks the mother about the reason and asks if her family/ people in her social circle describe it the same way or differently.
**Item 8: Method of dealing with problems and using the pre-existing methods of problem solving**<br>**1=Needs improvement**=Student does not ask the mother about how she deals with her problems or makes her own opinion about it. (For instance, why do you think it is beneficial or not)

**2=Done partially=** Student talks about dealing with problems and already existing solution but does not tell in a positive manner.<br>**3=Done well=** Student asks the mother about methods to deal with difficulties and then explains in appositive way.
**Item 9: Reviewing the mother's on going life incidents and circumstances and drawing their link with mother's psychological and social satisfaction**<br>**1=Needs improvement**=Student does not talk about incidents which create problems<br>**2=Done partially**=Student talks about life's incidents and circumstances/issues but does not connect the effects of unhealthy thoughts and moods with mother and child's satisfaction. (For instance when student shows picture A, B and C during the session).<br>**3=Done well**=Student talks about life incidents and circumstances and connects the effects of unhealthy thoughts and moods with mother and child's satisfaction (for example when she shows picture D, E and F during the session.)
**Item10: Reviewing the problems of mother's own health**<br>**1=Needs improvement=** student does not talk about the thoughts/concerns of the mother about mother's personal health.<br>**2=Done partially=** student talks about the thoughts/concerns of the mother about her personal health but does not draw the link between the unhealthy thoughts, mood, behaviours and their effects (with the help of picture and examples).<br>**3=Done well**= Student talks about the mother's thought/concerns regarding her personal health and wherever it is needed she draws a detailed link between the unhealthy thoughts, mood, behaviours and their effects (with the help of pictures and examples).
**Item11:Appropriate involvement of family members and other caretakers**<br>**1=Needs improvement=** when any family member is present: During the session, STUDENT ignores the family or only talks to the family members and ignores the mother. When no family member is present: Student does not talk about the family at all.<br>**2=Done partially=** when family members are present: Student talks to both, the mother and the child, but does not help them other and the family during the session to communicate with each other. When the family member is not present: STUDENT talks about the family involvement but does not take mother's view if she wants to involve the family or not.<br>**3=Done well=** when any family member is present: Student encourages and helps in the communication between the mother and the family member. When the family member is not present: Student asks mother about family involvement and guides her.

**Item12:Setting the goals mutually and talking about the mother's expectations**<br>**1=Needs improvement=** Student does not ask the mother about her goals and expectations regarding the treatment, or Student just tells the mother what to do without asking about her expectations.<br>**2=Done partially=** Student talks to mother about the goal/aim but does not discuss if this goal/ aim is achievable.<br>**3=Done well=** Student talks to the mother about the goal that what is achievable through treatment and what is not, and mother and student mutually decide the method/procedure of treatment.
**Item13: giving hope for achievable change**<br>**1=Needs improvement**= Student either does not give any hope (i.e you will never get well) or gives unrealistic hopes about the treatment and the betterment through it (i.e you will get well within few weeks and there will be no issues after that/in future)<br>**2=Done partially**= Student ambiguously tells the mother about what will happen during the treatment<br>**3=Done well**=STUDENT makes/ helps mother feel positive about the future and gives her achievable hopes about what can and cannot be achieved through the treatment. Student analyzes the mother's understanding of achievable change.
**Item14: Talking about mental health according to the level of understanding of local people**<br>**1=Needs Improvement**= Student uses complicated or embarrassing words while talking about mental health or she does not explain how the treatment would work.<br>**2=Done partially**= Student rarely uses any complicated words and does not use embarrassing words but she is unable to make the mother or the local people understand about the mother's mental health.<br>**3=Done well**= Student uses local proverbs and non-embarrassing language, according to the level of understanding of mother and local people, to talk about mental health and makes sure that the mother understands.
**Item15:Steps for problem solving: 1st step: Guiding how to recognize the unhealthy thinking; 2nd Step: Guiding how to replace the unhealthy thoughts with healthy thoughts; 3rd Step: Exercising and adopting the healthy thinking**<br>**1=Needs Improvement=** Student works with the mother to identify her unhealthy thinking.<br>**2=Done partially**= Student helps the mother to identify the unhealthy thoughts and changing it into healthy thinking.<br>**3=Done well**= Student helps the mother (1) To identify the unhealthy thoughts (2) To change the unhealthy thoughts into healthy thoughts (3) To decide about

the work to be done for adopting the healthy thinking according to the health chart.
**<u>Item16:Askingabout(mother's) opinion when a suggestion or advice is given while deciding the task</u>**<br>**1=Needs improvement**= Student tells the mother what to do without asking what she (mother) wants or what is easy/doable for her, or does not give any suggestion at all.<br>**2=Done partially=** Student explains to them other in a focused manner, for example, tells her to sleep for 7 hours at night while talking about rest chart but does not ask the mother if this suggestion is helpful for her.<br>**3=Done well=**When the mother asks, Student gives some advice and then asks about mother's opinion about those advices.
**Item17: Explaining and promoting (ensuring) privacy**<br>**1=Needs Improvement**= Student does not take care about privacy or does not talk according to the occasion.<br>**2=Done partially**= Student tells the mother that everything should be kept private but does not mention that things which could harm one's self or anyone else are exempted. Student talked about private matters even while the session was not done privately.<br>**3=Done well=**Student explains to the mother that this conversation should be kept private except for the things that could harm her or anyone else. STUDENT takes care of the fact that the session is private or not, and talks accordingly.
**<u>Item18: Causing harm to one's own self, causing harm to others, analysing the harm caused By others and mutually planning to deal with it.</u>**<br>**1=Needs Improvement**= Student does not ask about harming one's own self or anyone else.<br>**2=Done partially**=Student explains about causing harm to one's own self or others but does not help the mother in planning to deal with it.<br>**3=Done well**=Student talks about causing harm to one's own self or others and guides the mother for an appropriate strategy.
